# 1-Butyl-1-chloro-3-methyl-3*H*-2,1λ^4^-benzoxa­tellurole: crystal structure and Hirshfeld analysis

**DOI:** 10.1107/S2056989017003887

**Published:** 2017-03-24

**Authors:** Julio Zukerman-Schpector, Rodrigo Cunha, Álvaro T. Omori, Lucas Sousa Madureira, Edward R. T. Tiekink

**Affiliations:** a Departmento de Química, Universidade Federal de São Carlos, 13565-905 São Carlos, SP, Brazil; b Centro de Ciências Naturais e Humanas, Universidade Federal do ABC, Av. Dos Estados 5001, Bairro Bangu, CEP 09210-580 Santo André, SP, Brazil; c Centre for Crystalline Materials, School of Science and Technology, Sunway University, 47500 Bandar Sunway, Selangor Darul Ehsan, Malaysia

**Keywords:** crystal structure, tellurium, Hirshfeld surface analysis, heavy-atom chirality

## Abstract

Two independent mol­ecules comprise the asymmetric unit in the title benzoxatellurole compound, C_12_H_17_ClOTe. The mol­ecules, with the same chirality at the methine C atom, are connected into a loosely associated dimer by Te⋯O inter­actions, leading to a {⋯Te—O}_2_ core. The resultant C_2_ClO_2_ donor set approximates a square pyramid with the lone pair of electrons projected to occupy a position *trans* to the *n*-butyl substituent. Inter­estingly, the Te^IV^ atoms exhibit opposite chirality. The major difference between the independent mol­ecules relates to the conformation of the five-membered chelate rings, which is an envelope with the O atom being the flap, in one mol­ecule and is twisted about the O—C(methine) bond in the other. No directional inter­molecular inter­actions are noted in the mol­ecular packing beyond the aforementioned Te⋯O secondary bonding. The analysis of the Hirshfeld surface reveals the dominance of H⋯H contacts, *i.e*. contributing about 70% to the overall surface, and clearly differentiates the immediate crystalline environments of the two independent mol­ecules in terms of both H⋯H and H⋯Cl/Cl⋯H contacts.

## Abstract 2

Two chemically similar mol­ecules comprise the asymmetric unit and these are connected *via* Te⋯O secondary bonding. The coordination geometry for each mol­ecule is based on an octa­hedron with the lone-pair of electrons occupying a position opposite to the *n*-butyl group.

## Chemical context   

Tellurium is not the first element that comes to mind when considering the modern pharmacopoeia (Tiekink, 2012 ▸). However, investigations into pharmaceutical applications of compounds of this generally regarded as relatively non-toxic element (Nogueira *et al.*, 2004 ▸) date back to the times of Sir Alexander Fleming who tested the efficacy of potassium tellurite, K_2_[TeO_3_], against microbes, such as penicillin-insensitive bacteria (Fleming, 1932 ▸). It is in fact another salt, ammonium tri­chloro­(di­oxy­ethyl­ene-O,O′)tellurate, [NH_4_][(OCH_2_CH_2_O)TeCl_3_] (Albeck *et al.*, 1998 ▸), also known as AS-101, that has attracted the most attention as a potential tellurium-based pharmaceutical, being in clinical trials for the treatment of psoriasis (Halpert & Sredni, 2014 ▸). Other potential applications of AS-101 include its use as an anti-inflammatory agent (Brodsky, *et al.*, 2010 ▸), as a topical treatment for human papilloma virus (Friedman *et al.*, 2009 ▸) and its ability to inhibit angiogenesis (Sredni, 2012 ▸). The anti-cancer potential of tellurium compounds has also attracted attention (Seng & Tiekink, 2012 ▸; Silberman *et al.*, 2016 ▸). The cation in AS-101 has long been known to be a specific inhibitor of both papain and cathepsin B, *i.e*. cysteine proteases, by forming a covalent Te—S(cysteine) bond (Albeck *et al.*, 1998 ▸). Organotellurium compounds also inhibit cathepsin B (Cunha *et al.*, 2005 ▸) and docking studies confirm this hypothesis (Caracelli *et al.*, 2012 ▸, 2016 ▸). It was in this context that the title compound, (I), was prepared. Herein, the crystal and mol­ecular structures of (I) are described as well as an analysis of its Hirshfeld surface. Finally, a preliminary inhibition assay on (I) against cathepsin B has been performed.
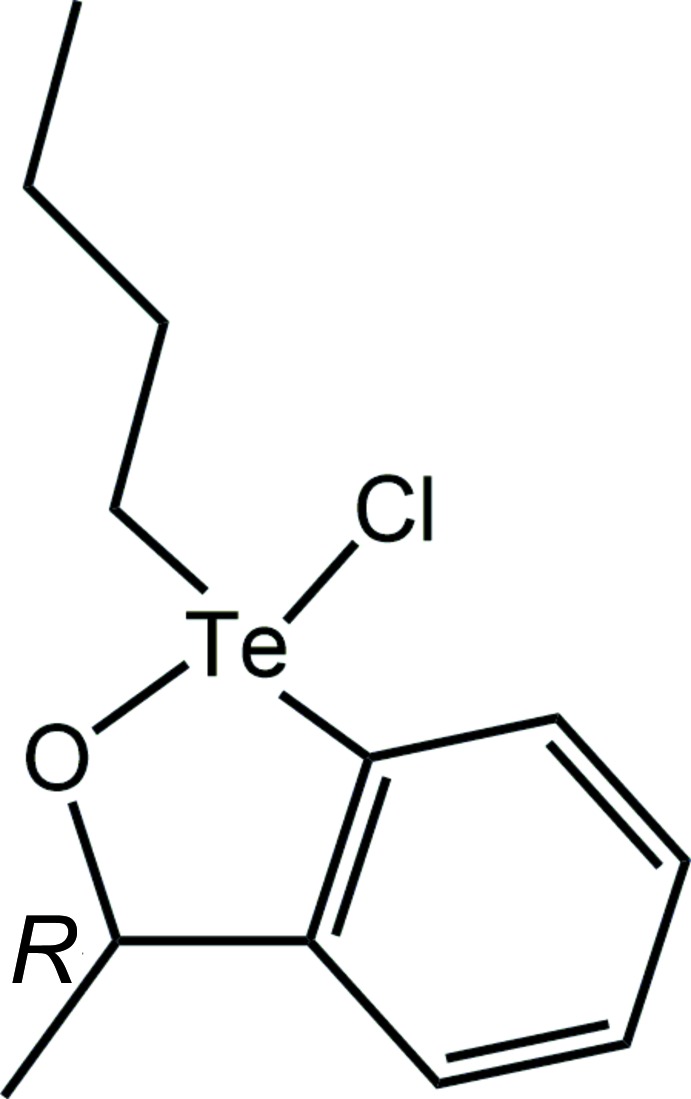


## Structural commentary   

**Figure 1:**
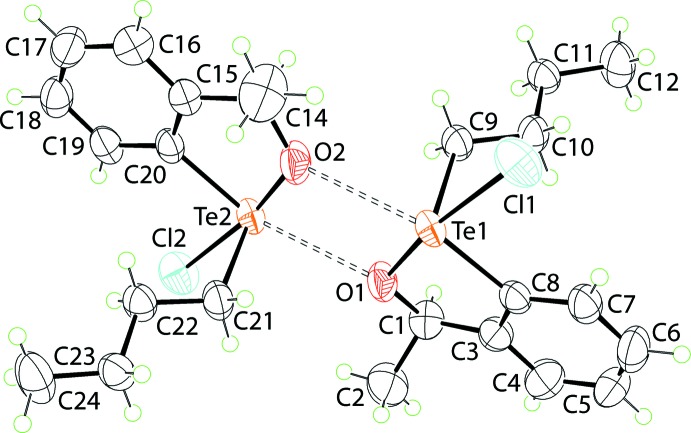
The mol­ecular structures of the two independent mol­ecules comprising the asymmetric unit of (I), showing the atom-labelling scheme and displacement ellipsoids at the 50% probability level. The mol­ecules associate *via* secondary Te⋯O bonding shown as dashed bonds.

The asymmetric unit of (I) comprises two independent mol­ecules, which are connected into a loosely associated dimer *via* secondary Te⋯O inter­actions, as shown in Fig. 1 ▸. The immediate geometry for the Te^IV^ atom in the Te1-containing mol­ecule is defined by chlorido, oxygen and carbon (within the oxatellurole ring) and *n*-butyl alpha-carbon atoms. While the bridging-O2 atom forms a significantly longer Te⋯O2 bond than the Te—O1 bond, Table 1 ▸, it must be included in the coordination geometry, which is then best described as being distorted square pyramidal. This arrangement accommodates a stereochemically active lone-pair of electrons in the position *trans* to the *n*-butyl group. The coordination geometry for the Te2-containing mol­ecule is essentially the same.

The bond lengths about the Te^IV^ atoms in the independent mol­ecules are similar, Table 1 ▸. However, the Te1—Cl1 bond length is longer by approximately 0.02 Å than the chemically equivalent Te—Cl2 bond. The three remaining ‘short’ bond lengths are equal within experimental error. The disparity in the Te—Cl bond lengths is probably compensated by the Te⋯O secondary bond, which is shorter, by approximately 0.03 Å, in the Te1-mol­ecule. The key pairs of bond angles for the mol­ecules are essentially the same with the major difference, *i.e*. 0.8°, seen in the Cl—Te—O_long_ angle. A distinguishing feature of the independent mol­ecules is noted in the conformation of the five-membered, chelate rings. Thus, in the Te1-mol­ecule, the chelate ring has the form of an envelope with the flap atom being the O1 atom [the O1 atom lies 0.254 (8) Å out of the plane through the remaining atoms; r.m.s. deviation = 0.0107 Å]. For the Te2-mol­ecule, the chelate ring is twisted about the O2—C13 bond, as seen in the Te2—O2—C13—C15 torsion angle of 12.1 (7)°.

**Figure 2:**
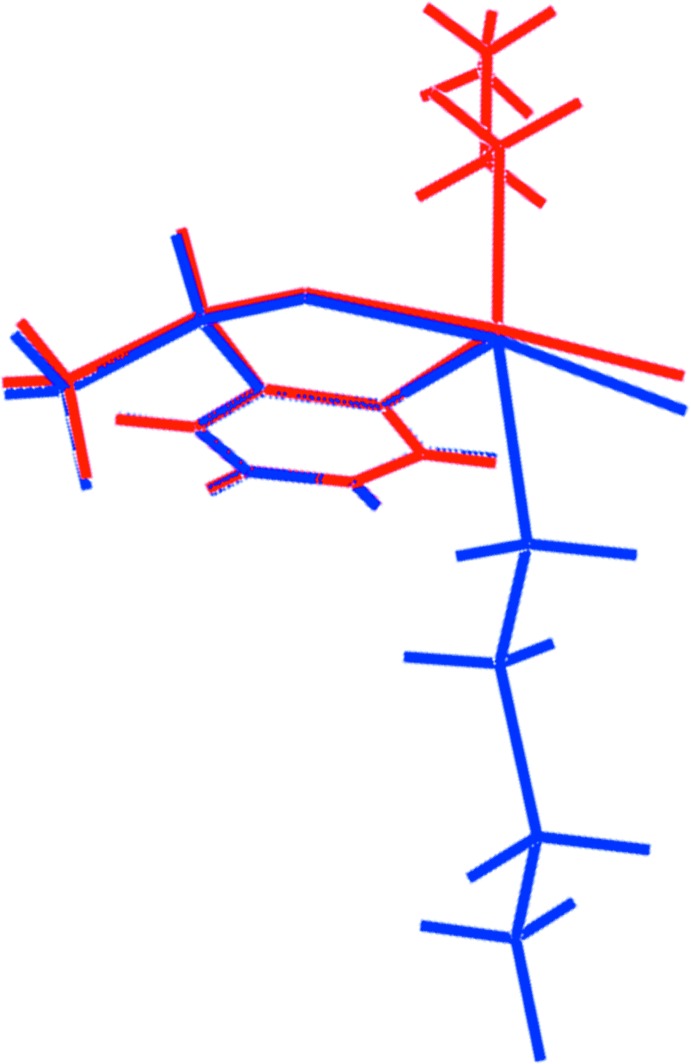
An overlay diagram of the Te1- and Te2-containing mol­ecules, shown as red and blue images, respectively. The mol­ecules have been overlapped so that the phenyl rings are coincident.

The central {⋯Te—O}_2_ core of the dimeric aggregate, Fig. 1 ▸, is almost planar (r.m.s. deviation = 0.0106 Å) and has the form of a parallelogram with distinctive edge lengths of approximately 2.0 and 3.0 Å, reflecting the disparity of the Te⋯O inter­actions. To a first approximation, the fused phenyl ring in each mol­ecule, (C3–C8) and (C13–C20), is co-planar with the core, forming dihedral angles of 14.2 (2) and 13.6 (3)°, respectively; the dihedral angle between the phenyl rings is 8.3 (3)°. As the *n*-butyl groups lie to either side of the dimeric aggregate, there is a suggestion that the independent mol­ecules are related across a pseudo centre of inversion. However, the configuration of the chiral-C2 and C13 atoms in the Te1- and Te-mol­ecules, respectively, is *R*. This is highlighted in the overlay diagram shown in Fig. 2 ▸. Also highlighted is that the tellurium atoms have opposite chirality. When projected down the Te—C(*n*-but­yl) bond, the chirality about the Te1 atom is *S* and that about Te2, *R*.

## Supra­molecular features   

**Figure 3:**
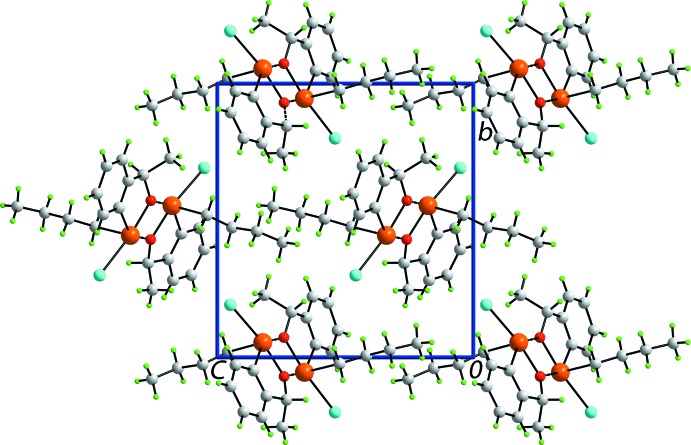
A view in projection down the *a* axis of the mol­ecular packing in (I).

Beyond the secondary Te⋯O secondary contacts, leading to dimeric aggregates, Fig. 1 ▸, no directional inter­actions, according to the criteria in *PLATON* (Spek, 2009 ▸), are apparent in the crystal of (I). A view of the unit-cell contents is shown in Fig. 3 ▸.

## Hirshfeld surface analysis   

**Figure 4:**
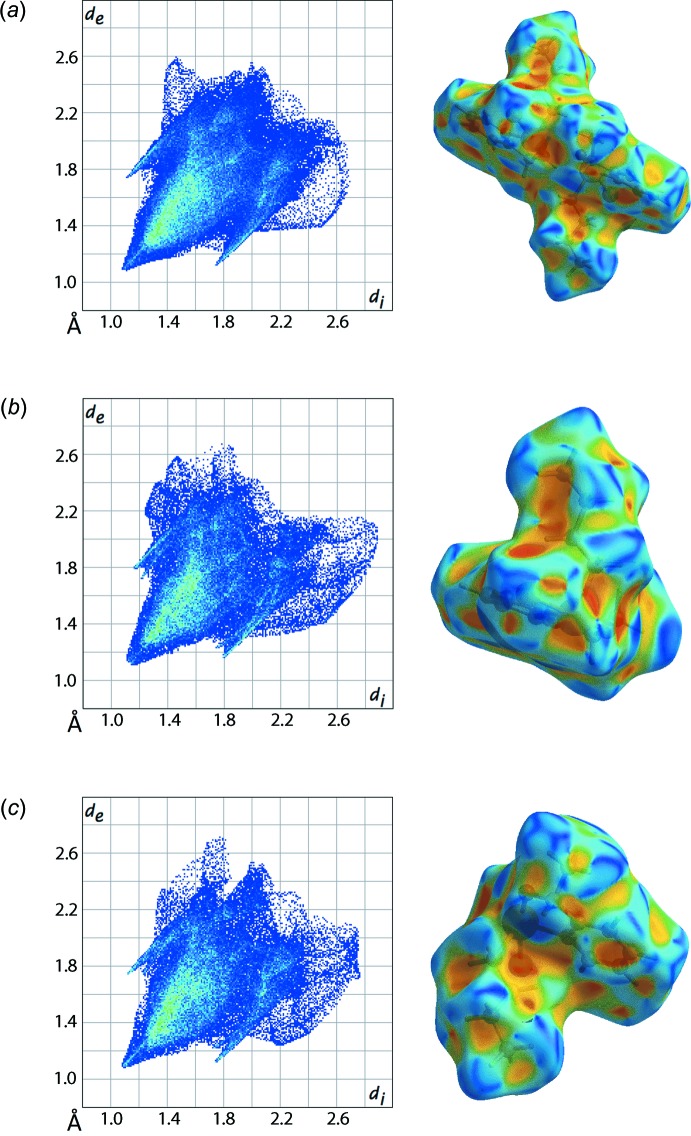
Two-dimensional fingerprint plots and shape index surface properties of the Hirshfeld surface analysis for (*a*) (I), (*b*) the Te1-mol­ecule in (I) and (*c*) the Te2-mol­ecule in (I).

An analysis of the Hirshfeld surface for (I) was conducted using protocols established earlier (Jotani *et al.*, 2016 ▸). The overall two-dimensional fingerprint plot for the asymmetric unit is shown in Fig. 4 ▸ *a* and those for the individual Te1- and Te2-containing mol­ecules are shown in Fig. 4 ▸ *b* and *c*. The shape-index surface properties are also illustrated in Fig. 4 ▸. These confirm the absence of significant directional inter­actions in the crystal.

**Figure 5:**
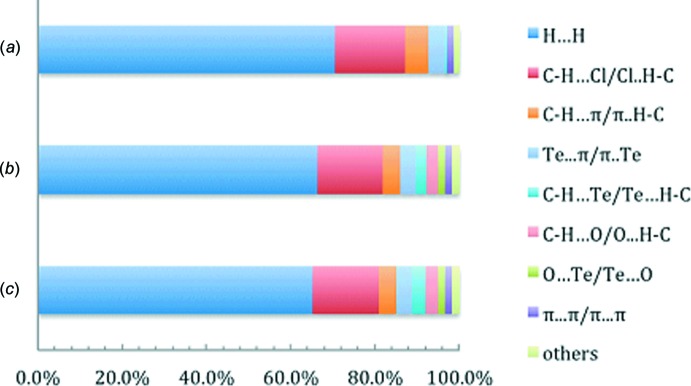
Charts of the relative percentage contributions of the inter­molecular contacts to the Hirshfeld surface area for (*a*) (I), (*b*) the Te1-mol­ecule in (I) and (*c*) the Te2-mol­ecule in (I).

Referring to Fig. 5 ▸ and Table 2 ▸, the Hirshfeld surface is dominated by H⋯H inter­actions, contributing around 70% to the overall surface of the asymmetric unit and about 65% for each independent mol­ecule. While not within the sum of the respective van de Waals radii, the C—H⋯Cl contacts make the next greatest contribution to the overall surface, *i.e. ca* 15%. Others inter­actions each contribute less than 5% to the Hirshfeld surface. It should be noted that the C—H⋯O contacts, Te⋯O secondary inter­actions and most of the C—H⋯Te contacts are formed between the two independent mol­ecules, thus they are overlapped and do not contribute to surface area of the asymmetric unit.

**Figure 6:**
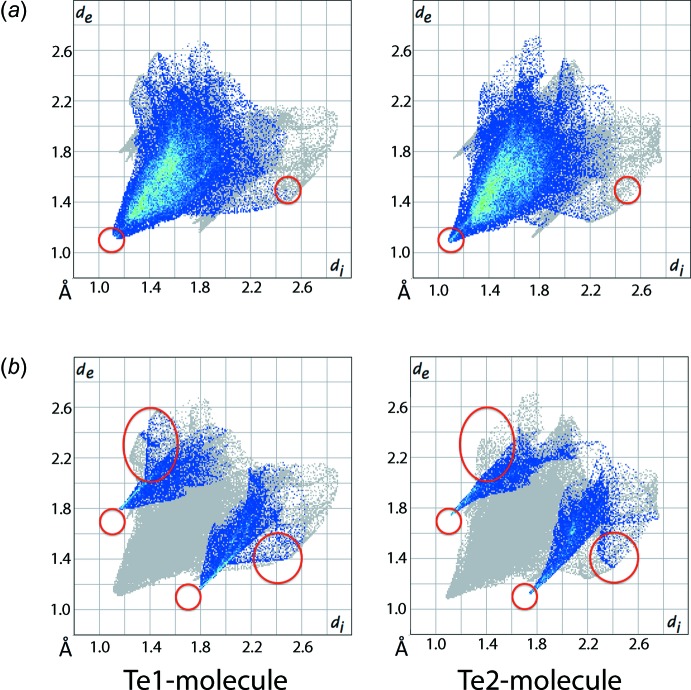
Two-dimensional fingerprint plots delineated into (*a*) H⋯H contacts and (*b*) H⋯Cl/Cl⋯H contacts for the Te1- and Te2-mol­ecules. The red circles highlight regions distinguishing the two independent mol­ecules.

The main differences between the surface areas of the independent mol­ecules are in the inter­actions of the type H⋯H and C—H⋯Cl. Referring to Fig. 6 ▸, the red circles on the fingerprint plots delineated into H⋯H, Fig. 6 ▸ *a* and H⋯Cl/Cl⋯H contacts, Fig. 6 ▸ *b*, highlight the distinctive features of the inter­actions for the two mol­ecules. For example, short H⋯H inter­actions for the Te2-mol­ecule, Fig. 6 ▸ *a*, occur at shorter distances that those of the Te1-mol­ecules. With regard to the H⋯Cl/Cl⋯H contacts, there is a wider spread at lower *d*_e_ + *d*_i_ for the Te1- *cf*. the Te2-mol­ecule.

## Database survey   

A search of the Cambridge Crystallographic Database (Groom *et al.*, 2016 ▸) reveals there are only 28 analogous structures featuring the TeOC_3_ donor set as in (I) without the bond type being specified. The number of ‘hits’ reduces to five with the inclusion of the aromatic ring in the side chain. Of the latter, the most closely related compound is 1-bromo-1-butyl-3*H*-2,1-benzoxatellurol (Maksimenko *et al.*, 1994 ▸), which is in fact very similar to (I), being derived from this by substituting the tellurium-bound chlorido atom with bromido and the removal of the methyl group. Here, the five-membered chelate ring is strictly planar.

## Inhibition of cathepsin B   

Compound (I) was screened for its ability to inhibit cathepsin B employing standard literature procedures (Cunha *et al.*, 2005 ▸). The determined value of the inhibition constant was 372 ± 40 *M* ^−1^ s^−1^, indicating some inhibitory potential, but not as potent as for other organotellurium(IV) compounds studied earlier (Cunha *et al.*, 2005 ▸).

## Synthesis and crystallization   

The compound was prepared following a literature procedure (Engman, 1984 ▸). The precursor chalcogenide, [2-(*R*)-MeCH(OH)]C_6_H_4_Te(*n*Bu) (1.52 g, 5 mmol), prepared as in the literature (Piovan *et al.*, 2011 ▸), was dissolved in dry di­chloro­methane (20 ml) and cooled to 253 K. To the stirred, cooled solution, sulfuryl chloride (0.4 ml, 5 mmol) dissolved in di­chloro­methane (5 ml) was added dropwise. The stirring was maintained for 20 minutes at 273 K and the solvent was then removed under reduced pressure. The oily product thus obtained was purified by crystallization from a mixture of dry benzene and pentane, yielding colourless crystals in 89% yield, m.p. 641.3–641.4 K. Analysis calculated for C_12_H_17_OClTe: C, 42.35, H, 5.03; Found C, 42.28, H, 4.98%. [α]_D_^26^ = +45.5° (CHCl_3_, *c* = 1.97). ^1^H (500.13 MHz, CDCl_3_, ppm) δ 8.20 (*d*, ^3^ *J* 7.6 Hz, 1H), 7.6–7.5 (*m*, 2H), 7.31 (*d*, ^3^ *J* 7.2 Hz, 1H), 5.59 (*q*, ^3^ *J* 6.3 Hz, 1H), 3.31 (*t*, ^3^ *J* 8.1 Hz, 2H), 1.90 (*quin*, ^3^ *J* 7.2 Hz, 2H), 1.59 (*d*, ^3^ *J* 6.45 Hz, 3H), 1.46 (*sext*, ^3^ *J* 7.4 Hz, 2H), 0.93 (*t*, ^3^ *J* 7.4 Hz, 3H). ^13^C (125 MHz, CDCl_3_, ppm) δ 148.1, 131.6, 131.2, 128.7, 127.8, 125.4, 75.5 (Br), 45.4, 28.4, 24.6, 23.7, 13.0. ^125^Te (157.85 MHz, CDCl_3_-*d*_6_, ppm) δ 847.2 (minor), 801.1 (major). ^125^Te (157.85 MHz, DMSO-*d*_6_, ppm) δ 1201.5 (minor), 1189.1 (major).

## Refinement details   

Crystal data, data collection and structure refinement details are summarized in Table 3 ▸. The carbon-bound H-atoms were placed in calculated positions (C—H = 0.93–0.98 Å) and were included in the refinement in the riding-model approximation, with *U*_iso_(H) set to 1.2–1.5*U*_eq_(C).

## Supplementary Material

Crystal structure: contains datablock(s) I, global. DOI: 10.1107/S2056989017003887/hg5483sup1.cif

Structure factors: contains datablock(s) I. DOI: 10.1107/S2056989017003887/hg5483Isup2.hkl

Supporting information file. DOI: 10.1107/S2056989017003887/hg5483Isup3.cml

CCDC reference: 1537011

Additional supporting information:  crystallographic information; 3D view; checkCIF report

## Figures and Tables

**Table 1:** Selected geometric parameters (Å, °)

Te1—Cl1	2.6137 (17)	Te2—Cl2	2.5944 (17)
Te1—O1	2.021 (4)	Te2—O2	2.010 (5)
Te1—C8	2.107 (6)	Te2—C20	2.108 (6)
Te1—C9	2.138 (5)	Te2—C21	2.136 (6)
Te1—O2	2.945 (4)	Te2—O1	2.977 (4)
			
Cl1—Te1—O1	171.04 (13)	Cl2—Te2—O2	170.22 (14)
O1—Te1—C8	80.4 (2)	O2—Te2—C20	80.5 (2)
C8—Te1—O2	145.0 (2)	C20—Te2—O1	145.38 (19)

**Table 2:** Percentage contributions of the different inter­molecular contacts to the Hirshfeld surface in (I), Te1-mol­ecule in (I) and Te2-mol­ecule in (I)

Contact	overall (I)	Te1-mol­ecule in (I)	Te-2 mol­ecule in (I)
H⋯H	70.3	65.1	66.2
H⋯C⋯l/Cl⋯H	16.6	15.7	15.4
H⋯π/π⋯H	5.5	4.1	4.2
Te⋯π/π⋯Te	4.0	3.7	3.6
H⋯Te/Te⋯H	0.4	3.3	2.6
H⋯O/O⋯H	0.0	2.9	2.9
O⋯Te/Te⋯O	0.0	1.7	1.6
π–π/π–π	1.7	1.5	1.5
Others	1.5	2.0	2.0

**Table 3:** Experimental details

Crystal data	
Chemical formula	C_12_H_17_ClOTe
*M*_r_	340.30
Crystal system, space group	Monoclinic, *P*2_1_
Temperature (K)	293
*a*, *b*, *c* (Å)	8.3663 (2), 13.0442 (4), 12.5363 (2)
β (°)	103.460 (2)
*V* (Å^3^)	1330.53 (6)
*Z*	4
Radiation type	Mo *K*α
μ (mm^−1^)	2.41
Crystal size (mm)	0.34 × 0.33 × 0.23

Data collection	
Diffractometer	Nonius KappaCCD
Absorption correction	Gaussian (Coppens *et al.*, 1965 ▸)
*T*_min_, *T*_max_	0.481, 0.550
No. of measured, independent and observed [*I* > 2σ(*I*)] reflections	9220, 5115, 4998
*R*_int_	0.061
(sin θ/λ)_max_ (Å^−1^)	0.650

Refinement	
*R*[*F* ^2^ > 2σ(*F* ^2^)], *wR*(*F* ^2^), *S*	0.027, 0.076, 1.02
No. of reflections	5115
No. of parameters	275
No. of restraints	1
H-atom treatment	H-atom parameters constrained
Δρ_max_, Δρ_min_ (e Å^−3^)	0.43, −0.82
Absolute structure	Flack *x* determined using 1908 quotients [(*I* ^+^)−(*I* ^−^)]/[(*I* ^+^)+(*I* ^−^)] (Parsons *et al.*, 2013 ▸)
Absolute structure parameter	−0.05 (3)

Computer programs: *COLLECT* (Nonius, 1998 ▸), *DENZO*/*SCALEPACK* (Otwinowski & Minor, 1997 ▸), *SIR2014* (Burla *et al.*, 2015 ▸), *SHELXL2014* (Sheldrick, 2015 ▸), *ORTEP-3 for Windows* (Farrugia, 2012 ▸), *QMol* (Gans & Shalloway, 2001 ▸), *DIAMOND* (Brandenburg, 2006 ▸) and *publCIF* (Westrip, 2010 ▸).

## supplementary crystallographic information

### Crystal data

**Table:**

C_12_H_17_ClOTe	*F*(000) = 664
*M**_r_* = 340.30	*D*_x_ = 1.699 Mg m^−^^3^
Monoclinic, *P*2_1_	Mo *K*α radiation, λ = 0.71073 Å
*a* = 8.3663 (2) Å	Cell parameters from 5903 reflections
*b* = 13.0442 (4) Å	θ = 1.0–27.5°
*c* = 12.5363 (2) Å	µ = 2.41 mm^−^^1^
β = 103.460 (2)°	*T* = 293 K
*V* = 1330.53 (6) Å^3^	Slab, colourless
*Z* = 4	0.34 × 0.33 × 0.23 mm

### Data collection

**Table:**

Nonius KappaCCD diffractometer	4998 reflections with *I* > 2σ(*I*)
CCD rotation images, thick slices scans	*R*_int_ = 0.061
Absorption correction: gaussian (Coppens *et al.*, 1965)	θ_max_ = 27.5°, θ_min_ = 2.5°
*T*_min_ = 0.481, *T*_max_ = 0.550	*h* = −10→8
9220 measured reflections	*k* = −15→16
5115 independent reflections	*l* = −16→13

### Refinement

**Table:**

Refinement on *F*^2^	Hydrogen site location: inferred from neighbouring sites
Least-squares matrix: full	H-atom parameters constrained
*R*[*F*^2^ > 2σ(*F*^2^)] = 0.027	*w* = 1/[σ^2^(*F*_o_^2^) + (0.0389*P*)^2^ + 0.6419*P*] where *P* = (*F*_o_^2^ + 2*F*_c_^2^)/3
*wR*(*F*^2^) = 0.076	(Δ/σ)_max_ < 0.001
*S* = 1.02	Δρ_max_ = 0.43 e Å^−^^3^
5115 reflections	Δρ_min_ = −0.82 e Å^−^^3^
275 parameters	Absolute structure: Flack *x* determined using 1908 quotients [(*I*^+^)-(*I*^-^)]/[(*I*^+^)+(*I*^-^)] (Parsons *et al.*, 2013)
1 restraint	Absolute structure parameter: −0.05 (3)

### Special details

**Table:**

Geometry. All esds (except the esd in the dihedral angle between two l.s. planes) are estimated using the full covariance matrix. The cell esds are taken into account individually in the estimation of esds in distances, angles and torsion angles; correlations between esds in cell parameters are only used when they are defined by crystal symmetry. An approximate (isotropic) treatment of cell esds is used for estimating esds involving l.s. planes.

### Fractional atomic coordinates and isotropic or equivalent isotropic displacement parameters (Å^2^)

**Table:**

	*x*	*y*	*z*	*U*_iso_*/*U*_eq_	
Te1	0.47914 (4)	0.44547 (2)	0.33996 (3)	0.04143 (11)	
Cl1	0.6469 (2)	0.30011 (15)	0.45946 (16)	0.0664 (4)	
O1	0.3777 (5)	0.5726 (4)	0.2598 (3)	0.0512 (10)	
C1	0.4636 (7)	0.6665 (4)	0.2894 (6)	0.0475 (12)	
H1	0.4101	0.7045	0.3388	0.057*	
C2	0.4470 (10)	0.7276 (6)	0.1836 (7)	0.075 (2)	
H2A	0.5040	0.6927	0.1361	0.112*	
H2B	0.4936	0.7946	0.2005	0.112*	
H2C	0.3328	0.7340	0.1476	0.112*	
C3	0.6380 (6)	0.6452 (5)	0.3476 (5)	0.0458 (11)	
C4	0.7594 (8)	0.7216 (6)	0.3717 (7)	0.0652 (17)	
H4	0.7345	0.7880	0.3462	0.078*	
C5	0.9147 (8)	0.6994 (8)	0.4326 (7)	0.072 (2)	
H5	0.9941	0.7505	0.4491	0.086*	
C6	0.9523 (7)	0.5984 (9)	0.4698 (6)	0.075 (3)	
H6	1.0570	0.5831	0.5113	0.090*	
C7	0.8366 (6)	0.5227 (6)	0.4454 (5)	0.0533 (15)	
H7	0.8622	0.4560	0.4695	0.064*	
C8	0.6800 (6)	0.5467 (5)	0.3842 (4)	0.0416 (10)	
C9	0.3754 (6)	0.4771 (5)	0.4770 (4)	0.0474 (13)	
H9A	0.2963	0.5322	0.4567	0.057*	
H9B	0.3154	0.4168	0.4909	0.057*	
C10	0.4927 (6)	0.5063 (6)	0.5816 (5)	0.0510 (14)	
H10A	0.5533	0.5668	0.5691	0.061*	
H10B	0.5711	0.4511	0.6040	0.061*	
C11	0.4067 (7)	0.5281 (5)	0.6735 (4)	0.0479 (14)	
H11A	0.3329	0.5858	0.6524	0.057*	
H11B	0.3405	0.4691	0.6824	0.057*	
C12	0.5220 (8)	0.5516 (9)	0.7815 (5)	0.0674 (18)	
H12A	0.6033	0.4984	0.7992	0.101*	
H12B	0.4614	0.5553	0.8376	0.101*	
H12C	0.5753	0.6161	0.7767	0.101*	
Te2	0.01522 (4)	0.55421 (2)	0.17723 (3)	0.04226 (11)	
Cl2	−0.1556 (2)	0.69208 (15)	0.05090 (16)	0.0666 (4)	
O2	0.1199 (5)	0.4303 (4)	0.2604 (4)	0.0583 (11)	
C13	0.0191 (7)	0.3453 (5)	0.2705 (5)	0.0530 (13)	
H13	0.0032	0.3452	0.3455	0.064*	
C14	0.1031 (10)	0.2471 (6)	0.2538 (9)	0.083 (3)	
H14A	0.1177	0.2445	0.1801	0.125*	
H14B	0.0371	0.1901	0.2661	0.125*	
H14C	0.2085	0.2439	0.3045	0.125*	
C15	−0.1500 (7)	0.3589 (5)	0.1920 (5)	0.0498 (13)	
C16	−0.2697 (8)	0.2822 (6)	0.1731 (6)	0.0627 (16)	
H16	−0.2486	0.2191	0.2080	0.075*	
C17	−0.4201 (8)	0.3008 (7)	0.1021 (6)	0.0646 (19)	
H17	−0.4989	0.2492	0.0881	0.077*	
C18	−0.4547 (7)	0.3946 (7)	0.0519 (5)	0.0585 (17)	
H18	−0.5568	0.4059	0.0048	0.070*	
C19	−0.3397 (7)	0.4717 (5)	0.0709 (5)	0.0508 (14)	
H19	−0.3636	0.5356	0.0380	0.061*	
C20	−0.1858 (5)	0.4524 (5)	0.1407 (4)	0.0413 (10)	
C21	0.1184 (6)	0.5194 (6)	0.0408 (5)	0.0546 (16)	
H21A	0.1644	0.5819	0.0185	0.066*	
H21B	0.2084	0.4717	0.0652	0.066*	
C22	0.0033 (7)	0.4747 (5)	−0.0574 (5)	0.0485 (14)	
H22A	−0.0914	0.5194	−0.0794	0.058*	
H22B	−0.0352	0.4088	−0.0380	0.058*	
C23	0.0837 (7)	0.4607 (6)	−0.1542 (5)	0.0506 (13)	
H23A	0.1271	0.5262	−0.1710	0.061*	
H23B	0.1753	0.4137	−0.1330	0.061*	
C24	−0.0311 (9)	0.4209 (8)	−0.2549 (6)	0.079 (3)	
H24A	−0.0737	0.3556	−0.2392	0.118*	
H24B	0.0266	0.4130	−0.3122	0.118*	
H24C	−0.1203	0.4682	−0.2780	0.118*	

### Atomic displacement parameters (Å^2^)

**Table:**

	*U*^11^	*U*^22^	*U*^33^	*U*^12^	*U*^13^	*U*^23^
Te1	0.04719 (18)	0.0443 (2)	0.02938 (15)	0.00273 (13)	0.00198 (11)	−0.00116 (15)
Cl1	0.0837 (11)	0.0546 (9)	0.0580 (10)	0.0195 (8)	0.0106 (8)	0.0102 (7)
O1	0.0467 (18)	0.050 (3)	0.047 (2)	0.0016 (17)	−0.0085 (15)	0.0090 (18)
C1	0.052 (3)	0.033 (2)	0.055 (3)	0.004 (2)	0.007 (2)	−0.003 (3)
C2	0.079 (4)	0.060 (4)	0.076 (5)	0.000 (3)	0.000 (4)	0.025 (4)
C3	0.047 (2)	0.049 (3)	0.042 (3)	−0.001 (2)	0.012 (2)	−0.003 (2)
C4	0.065 (4)	0.059 (4)	0.070 (5)	−0.014 (3)	0.012 (3)	−0.002 (3)
C5	0.046 (3)	0.099 (6)	0.068 (5)	−0.022 (3)	0.009 (3)	−0.004 (4)
C6	0.034 (3)	0.133 (8)	0.054 (4)	0.001 (4)	0.005 (2)	−0.014 (5)
C7	0.039 (2)	0.078 (5)	0.042 (3)	0.010 (2)	0.008 (2)	0.001 (3)
C8	0.041 (2)	0.055 (3)	0.030 (2)	0.003 (2)	0.0091 (17)	0.000 (2)
C9	0.040 (2)	0.067 (4)	0.034 (2)	−0.001 (2)	0.0074 (19)	−0.004 (2)
C10	0.039 (2)	0.077 (4)	0.037 (3)	0.000 (2)	0.008 (2)	−0.010 (3)
C11	0.047 (2)	0.061 (4)	0.036 (3)	0.002 (2)	0.010 (2)	−0.002 (2)
C12	0.062 (3)	0.096 (5)	0.043 (3)	0.002 (4)	0.008 (2)	−0.022 (4)
Te2	0.04777 (18)	0.0432 (2)	0.03135 (16)	0.00212 (13)	0.00019 (12)	−0.00352 (14)
Cl2	0.0768 (10)	0.0535 (9)	0.0646 (10)	0.0164 (8)	0.0063 (8)	0.0099 (8)
O2	0.052 (2)	0.056 (3)	0.055 (2)	−0.001 (2)	−0.0130 (17)	0.008 (2)
C13	0.060 (3)	0.056 (3)	0.037 (3)	0.001 (3)	−0.001 (2)	0.001 (3)
C14	0.073 (4)	0.058 (4)	0.110 (7)	0.012 (3)	0.003 (4)	−0.004 (4)
C15	0.052 (3)	0.054 (3)	0.041 (3)	−0.005 (2)	0.005 (2)	0.001 (2)
C16	0.063 (3)	0.065 (4)	0.059 (4)	−0.009 (3)	0.013 (3)	0.002 (3)
C17	0.055 (3)	0.081 (5)	0.059 (4)	−0.021 (3)	0.013 (3)	−0.017 (4)
C18	0.038 (3)	0.087 (5)	0.050 (3)	−0.003 (3)	0.009 (2)	−0.014 (3)
C19	0.047 (3)	0.067 (4)	0.037 (3)	0.010 (2)	0.006 (2)	0.000 (2)
C20	0.040 (2)	0.054 (3)	0.029 (2)	0.002 (2)	0.0053 (16)	−0.006 (2)
C21	0.042 (2)	0.083 (5)	0.037 (3)	−0.001 (3)	0.005 (2)	−0.010 (3)
C22	0.046 (2)	0.061 (4)	0.039 (3)	0.003 (2)	0.010 (2)	−0.008 (2)
C23	0.049 (2)	0.062 (4)	0.041 (3)	0.005 (2)	0.012 (2)	0.001 (3)
C24	0.068 (4)	0.121 (9)	0.045 (3)	0.015 (4)	0.008 (3)	−0.019 (4)

### Geometric parameters (Å, º)

**Table:**

Te1—Cl1	2.6137 (17)	C11—H11A	0.9700
Te1—O1	2.021 (4)	C11—H11B	0.9700
Te1—C8	2.107 (6)	C12—H12A	0.9600
Te1—C9	2.138 (5)	C12—H12B	0.9600
Te1—O2	2.945 (4)	C12—H12C	0.9600
Te2—Cl2	2.5944 (17)	O2—C13	1.416 (8)
Te2—O2	2.010 (5)	C13—C14	1.499 (10)
Te2—C20	2.108 (6)	C13—C15	1.534 (7)
Te2—C21	2.136 (6)	C13—H13	0.9800
Te2—O1	2.977 (4)	C14—H14A	0.9600
O1—C1	1.424 (7)	C14—H14B	0.9600
C1—C3	1.497 (7)	C14—H14C	0.9600
C1—C2	1.526 (10)	C15—C20	1.379 (9)
C1—H1	0.9800	C15—C16	1.396 (9)
C2—H2A	0.9600	C16—C17	1.383 (9)
C2—H2B	0.9600	C16—H16	0.9300
C2—H2C	0.9600	C17—C18	1.376 (12)
C3—C8	1.382 (9)	C17—H17	0.9300
C3—C4	1.405 (9)	C18—C19	1.374 (10)
C4—C5	1.376 (10)	C18—H18	0.9300
C4—H4	0.9300	C19—C20	1.401 (7)
C5—C6	1.408 (14)	C19—H19	0.9300
C5—H5	0.9300	C21—C22	1.494 (7)
C6—C7	1.367 (11)	C21—H21A	0.9700
C6—H6	0.9300	C21—H21B	0.9700
C7—C8	1.391 (7)	C22—C23	1.529 (7)
C7—H7	0.9300	C22—H22A	0.9700
C9—C10	1.493 (7)	C22—H22B	0.9700
C9—H9A	0.9700	C23—C24	1.490 (9)
C9—H9B	0.9700	C23—H23A	0.9700
C10—C11	1.521 (7)	C23—H23B	0.9700
C10—H10A	0.9700	C24—H24A	0.9600
C10—H10B	0.9700	C24—H24B	0.9600
C11—C12	1.500 (8)	C24—H24C	0.9600
			
Cl1—Te1—O1	171.04 (13)	C10—C11—H11A	108.8
O1—Te1—C8	80.4 (2)	C12—C11—H11B	108.8
C8—Te1—O2	145.0 (2)	C10—C11—H11B	108.8
Cl2—Te2—O2	170.22 (14)	H11A—C11—H11B	107.7
O2—Te2—C20	80.5 (2)	C11—C12—H12A	109.5
C20—Te2—O1	145.38 (19)	C11—C12—H12B	109.5
O1—Te1—O2	66.99 (14)	H12A—C12—H12B	109.5
O1—Te1—C9	92.2 (2)	C11—C12—H12C	109.5
C8—Te1—C9	96.7 (2)	H12A—C12—H12C	109.5
C8—Te1—Cl1	90.85 (17)	H12B—C12—H12C	109.5
C9—Te1—Cl1	86.78 (17)	C13—O2—Te2	118.6 (3)
C9—Te1—O2	73.25 (17)	C13—O2—Te1	127.2 (3)
Cl1—Te1—O2	121.01 (11)	Te2—O2—Te1	114.1 (2)
C1—O1—Te1	116.6 (3)	O2—C13—C14	110.4 (5)
C1—O1—Te2	124.9 (3)	O2—C13—C15	109.4 (5)
Te1—O1—Te2	112.50 (18)	C14—C13—C15	113.6 (6)
O2—Te2—C21	92.1 (2)	O2—C13—H13	107.7
C20—Te2—C21	98.2 (2)	C14—C13—H13	107.7
C20—Te2—Cl2	90.34 (16)	C15—C13—H13	107.7
C21—Te2—Cl2	85.79 (19)	C13—C14—H14A	109.5
O2—Te2—O1	66.36 (14)	C13—C14—H14B	109.5
C21—Te2—O1	74.15 (17)	H14A—C14—H14B	109.5
Cl2—Te2—O1	121.88 (10)	C13—C14—H14C	109.5
O1—C1—C3	110.1 (5)	H14A—C14—H14C	109.5
O1—C1—C2	106.5 (6)	H14B—C14—H14C	109.5
C3—C1—C2	113.7 (5)	C20—C15—C16	119.0 (5)
O1—C1—H1	108.8	C20—C15—C13	118.0 (5)
C3—C1—H1	108.8	C16—C15—C13	123.0 (6)
C2—C1—H1	108.8	C17—C16—C15	119.5 (7)
C1—C2—H2A	109.5	C17—C16—H16	120.3
C1—C2—H2B	109.5	C15—C16—H16	120.3
H2A—C2—H2B	109.5	C16—C17—C18	120.9 (7)
C1—C2—H2C	109.5	C16—C17—H17	119.5
H2A—C2—H2C	109.5	C18—C17—H17	119.5
H2B—C2—H2C	109.5	C19—C18—C17	120.5 (6)
C8—C3—C4	118.3 (5)	C19—C18—H18	119.8
C8—C3—C1	118.5 (5)	C17—C18—H18	119.8
C4—C3—C1	123.1 (6)	C18—C19—C20	118.7 (6)
C5—C4—C3	120.8 (8)	C18—C19—H19	120.6
C5—C4—H4	119.6	C20—C19—H19	120.6
C3—C4—H4	119.6	C15—C20—C19	121.3 (6)
C4—C5—C6	119.4 (7)	C15—C20—Te2	112.3 (3)
C4—C5—H5	120.3	C19—C20—Te2	126.4 (5)
C6—C5—H5	120.3	C22—C21—Te2	116.1 (4)
C7—C6—C5	120.6 (6)	C22—C21—H21A	108.3
C7—C6—H6	119.7	Te2—C21—H21A	108.3
C5—C6—H6	119.7	C22—C21—H21B	108.3
C6—C7—C8	119.2 (7)	Te2—C21—H21B	108.3
C6—C7—H7	120.4	H21A—C21—H21B	107.4
C8—C7—H7	120.4	C21—C22—C23	112.5 (5)
C3—C8—C7	121.7 (6)	C21—C22—H22A	109.1
C3—C8—Te1	111.7 (4)	C23—C22—H22A	109.1
C7—C8—Te1	126.5 (5)	C21—C22—H22B	109.1
C10—C9—Te1	116.6 (3)	C23—C22—H22B	109.1
C10—C9—H9A	108.1	H22A—C22—H22B	107.8
Te1—C9—H9A	108.1	C24—C23—C22	113.5 (5)
C10—C9—H9B	108.1	C24—C23—H23A	108.9
Te1—C9—H9B	108.1	C22—C23—H23A	108.9
H9A—C9—H9B	107.3	C24—C23—H23B	108.9
C9—C10—C11	112.5 (4)	C22—C23—H23B	108.9
C9—C10—H10A	109.1	H23A—C23—H23B	107.7
C11—C10—H10A	109.1	C23—C24—H24A	109.5
C9—C10—H10B	109.1	C23—C24—H24B	109.5
C11—C10—H10B	109.1	H24A—C24—H24B	109.5
H10A—C10—H10B	107.8	C23—C24—H24C	109.5
C12—C11—C10	113.8 (5)	H24A—C24—H24C	109.5
C12—C11—H11A	108.8	H24B—C24—H24C	109.5
			
Te1—O1—C1—C3	18.3 (7)	Te2—O2—C13—C14	137.8 (6)
Te2—O1—C1—C3	168.9 (3)	Te1—O2—C13—C14	−39.0 (7)
Te1—O1—C1—C2	142.1 (4)	Te2—O2—C13—C15	12.1 (7)
Te2—O1—C1—C2	−67.4 (6)	Te1—O2—C13—C15	−164.8 (4)
O1—C1—C3—C8	−13.3 (8)	O2—C13—C15—C20	−10.0 (8)
C2—C1—C3—C8	−132.8 (6)	C14—C13—C15—C20	−133.9 (7)
O1—C1—C3—C4	169.8 (6)	O2—C13—C15—C16	171.9 (6)
C2—C1—C3—C4	50.4 (9)	C14—C13—C15—C16	48.1 (9)
C8—C3—C4—C5	−1.8 (10)	C20—C15—C16—C17	1.2 (10)
C1—C3—C4—C5	175.1 (7)	C13—C15—C16—C17	179.2 (7)
C3—C4—C5—C6	0.9 (12)	C15—C16—C17—C18	−1.6 (11)
C4—C5—C6—C7	0.3 (12)	C16—C17—C18—C19	0.4 (11)
C5—C6—C7—C8	−0.6 (10)	C17—C18—C19—C20	1.1 (9)
C4—C3—C8—C7	1.5 (9)	C16—C15—C20—C19	0.4 (9)
C1—C3—C8—C7	−175.5 (5)	C13—C15—C20—C19	−177.7 (5)
C4—C3—C8—Te1	179.6 (5)	C16—C15—C20—Te2	−178.1 (5)
C1—C3—C8—Te1	2.6 (6)	C13—C15—C20—Te2	3.7 (7)
C6—C7—C8—C3	−0.3 (9)	C18—C19—C20—C15	−1.6 (8)
C6—C7—C8—Te1	−178.1 (5)	C18—C19—C20—Te2	176.8 (4)
Te1—C9—C10—C11	179.5 (5)	Te2—C21—C22—C23	175.1 (5)
C9—C10—C11—C12	176.6 (8)	C21—C22—C23—C24	−177.3 (7)
